# MPXV replication and drug resistance in CNS of advanced HIV case at autopsy

**DOI:** 10.21203/rs.3.rs-9282876/v1

**Published:** 2026-04-09

**Authors:** Daniel Chertow, Maria Charles, Sofia Romero Ferrufino, Sabrina Ramelli, Shane Gallogly, Claude Kwe Yinda, Divya Ahuja, Edwin Hayes, Morgan Pizzuti, Kevin Vannella, Craig Martens, Franziska Kaiser, Andrew Platt, Martha Quezado, Christopher Morris, Vincent Munster, Stephen Hewitt

**Affiliations:** NIH; NIH; NIH; National Institutes of Health; NIH; National Institute of Allergy and Infectious Diseases; Prisma Health; Prisma Health; Prisma Health; National Institutes of Health; NIH; National Institute of Allergy and Infectious Diseases; NIH; National Institutes of Health; NIH; NIH; National Institutes of Health

## Abstract

Monkeypox virus (MPXV) clade IIb caused an outbreak of >100,000 cases globally, with highest disease burden in people living with advanced human immunodeficiency virus (HIV), those with CD4 count <200 cells/mm3. We performed an extensive autopsy on a fatal case of mpox in a person living with advanced HIV-1, who achieved HIV suppression but poor immune reconstitution despite > 6 months antiretroviral therapy and had progressive necrotizing mucocutaneous mpox disease despite multiple prolonged mpox-targeted therapies. We quantified MPXV DNA across 76 tissues and observed a high burden in skin and mucosal tissue and a lower burden in all central nervous system (CNS) tissues tested. However, we detected replication-competent MPXV in multiple CNS tissues and confirmed neuronal and glial cell infection using RNA-scope in situ hybridization. MPVX sequencing from body and brain tissues revealed widespread tecovirimat resistance-associated variants. This case proves that replication-competent MPXV and tecovirimat resistance-associated isolates can infect and persist in the CNS of people with advanced HIV thus informing future therapeutic design.

## Introduction

In 2022 monkeypox virus (MPXV) clade IIb caused a global outbreak with an estimated 124,000 cases,^1^ 38–50% occurring in people living with human immunodeficiency virus (HIV).^2^ While mpox, the disease caused by MPXV infection, is typically self-limited, patients with advanced HIV (CD4 count < 200 cells/mm^3^) experience severe, prolonged illness with necrotizing mucocutaneous lesions, bacterial coinfections, respiratory complications, and high mortality.^2^ Autopsies of immunocompromised patients with mpox show multiorgan infection, tissue necrosis, and vasculopathy,^3,4^ but the absolute burden, replication-competence, and distribution of drug resistant MPXV across tissues including the brain has not been described. MPXV clade IIb sequencing analyses indicate faster evolution than other Orthopoxviruses, and mutational biases suggest apolipoprotein B mRNA editing enzyme, catalytic polypeptide-like 3 (APOBEC3)-mediated intrahost evolution.^5^ Longitudinal skin or mucosal swab sampling of patients with advanced HIV and prolonged MPXV shedding reveal site-specific single nucleotide variants,^6^ including mutations in the F13L gene encoding VP37, associated with tecovirimat resistance.^7,8^ Here, we report widespread MPXV tissue distribution, cellular tropism, replication-competence, and tecovirimat resistance-associated variants throughout the body including brain at autopsy of a patient with advanced HIV and mpox for >7 months. We quantified MPXV DNA by droplet digital polymerase chain reaction (ddPCR) from 40 distinct anatomic sites, confirmed cellular tropism by RNAscope in situ hybridization (ISH), and isolated replication-competent MPXV from 15 distinct anatomic sites including multiple brain regions. MPXV full genome sequencing revealed limited viral diversity across anatomic compartments with widespread distribution of variants carrying tecovirimat resistance-associated mutations.

## Methods

### MPXV DNA quantification and sequencing

Following consent of legal next-of-kin, the autopsy was performed at the National Institutes of Health Clinical Center as previously described.^9, 10^ Total nucleic acid was extracted from RNAlater (Invitrogen, Cat No. AM7020)-preserved tissues using the DNeasy Blood and Tissue Kit (Qiagen, Cat No. 69516) per manufacturer’s instructions eluted in 100uL nuclease-free water. Three distinct MPXV gene targets in each sample were quantified using the QX600 AutoDG ddPCR System (Bio-Rad) (supplemental methods). DNA sequencing libraries were prepared, sequenced, analyzed, and visualized as described in supplemental methods.

### MPXV isolation and cellular localization

Frozen tissue sections were homogenized, diluted supernatants were cultured in Vero E6 cells at 37°C for five days in DMEM2, plates were scored for cytopathic effect, and supernatant and cell monolayers were passaged a second time. Replication-competent MPXV was validated by a reduction in quantitative real-time polymerase chain reaction (qRT-PCR) cycle threshold (Ct) from first to second passage (supplemental methods). Mpox ISH was performed as previously described^10^ with RNAscopeTM Probe-V-Monkeypox (Cat No. 534671) and modifications.^11^

## Results

### Clinical case and pathological findings

A 38-year-old African American male living with HIV-1, without prior mpox vaccination, and a recent history of Guillain-Barre Syndrome and neurosyphilis treated with intravenous immunoglobulin (IVIG), IV penicillin, and initiation of antiretroviral therapy (ART) with dolutegravir and emtricitabine/tenofovir alafenamide developed facial cutaneous lesions, confirmed to be mpox by RT-PCR of lesional swabs, in November 2022. Due to progressive purulent lesions on the face and groin he was hospitalization in January 2023 at which time his CD4 count was 137 cells/mm^3^ and HIV viral load was 30 copies/ml, down from 1.3e6 copies/ml prior to initiating ART. Initial mpox-targeted therapy included oral tecovirimat 600mg every 12 hours for 14-days and one dose of IV cidofovir 375mg and probenecid. During and following initial mpox-targeted therapy, new lesions developed. Therefore, over the subsequent five months of hospitalization he received an additional 18 weeks of oral or iv tecovirimat (>20 weeks total), nine more doses of IV cidofovir and probenecid (10 doses total), four doses of IV vaccinia immune globulin (VIGIV, CNJ-016, EA-IND) 9,000 units/kg, and two weeks of oral brincidofovir 200 mg weekly. Despite these treatments his lesions enlarged, ulcerated, and remained mpox RT-PCR positive. His clinical condition progressively deteriorated with failure to thrive and inability to tolerate oral intake. By the end of June 2023, the patient entered hospice with a CD4 count of 153 cells/mm^3^ and an undetectable viral load and died eight days later. Autopsy findings after a 30-hour post-mortem interval revealed a cachectic male with extensive cutaneous lesions in various stages over the face, torso, limbs, and groin including desquamated skin, crusted pustules, and hypopigmented scars. No pre- or perimortem blood sample was available for testing. Other notable pathological findings included pulmonary edema, focal bland pulmonary hemorrhage, ischemic changes in liver zone 3, left-shifted granulocytic hyperplasia and plasmacytosis in bone marrow, and acute tubular injury in kidneys.

### Widespread MPXV infection, cellular tropism, and replication-competence

**Figure 1:**
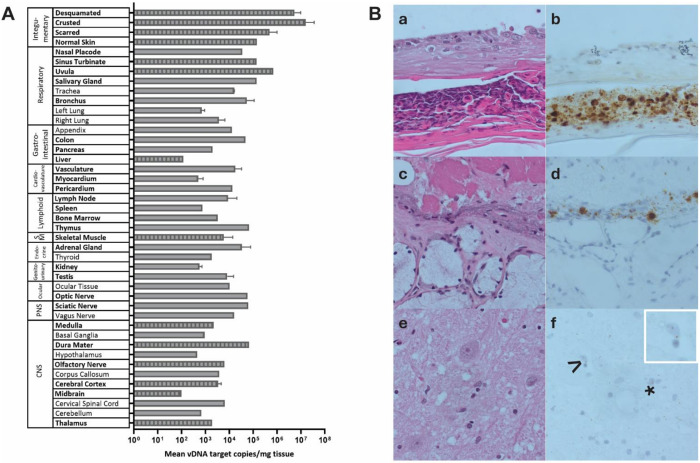
MPXV DNA quantification, replication-competence, and cellular localization. **A.** The bar graph illustrates the mean of VP39, A22R, and A13L MPXV gene copy/mg tissue measured via droplet digital PCR. Error bars indicate mean ± 1 SD for compartments with multiple tissues. Bolded tissues were evaluated for replication-competence in Vero E6 cell culture and hash marks indicate culture positive tissues. SM, skeletal muscle; PNS, peripheral nervous system; CNS, central nervous system. **B.** H&E **(a, c, e)** histology and matching RNA in situ hybridization **(b, d, f)** for detection of MPOX in skin **(a and b)** with the presence of virus in epidermal layer, with acute inflammatory cells absent in keratinized epithelium; uvula **(d and e)**with presence of virus in a cleft of necrotic epithelium; and midbrain **(e and f)** with presence of virus in a neuron (> and white box inset) and glial cells (*), without cytopathic features.

We detected MPXV DNA in 76 samples tested across 40 distinct anatomic sites from all major organ systems with mean copies/mg of tissue ranging from 1e2 to 7e7 (Fig. 1a). Highest burden of MPXV DNA was detected in skin and naso-oropharyngeal tissues with > 1e5 copies/mg detected in sinus turbinate, uvula, and salivary gland. MPXV DNA was detected in all central nervous system (CNS) tissues evaluated including cerebral cortex, cerebellum, brainstem, thalamus, and spinal cord, among others. We confirmed MPXV cellular localization by ISH in tissues throughout the body including in neurons and glial cells in the brain (Fig. 1b, Table S1). We confirmed replication-competent MPXV in Vero E6 cell culture from 12 of 33 tissues tested including skin, sinus turbinate, olfactory nerve, dura mater, frontal lobe, medulla, and midbrain (Fig. 1b, Table S2).

### Tecovirimat resistance-associated MPXV variants detected throughout the body including brain

**Figure 2:**
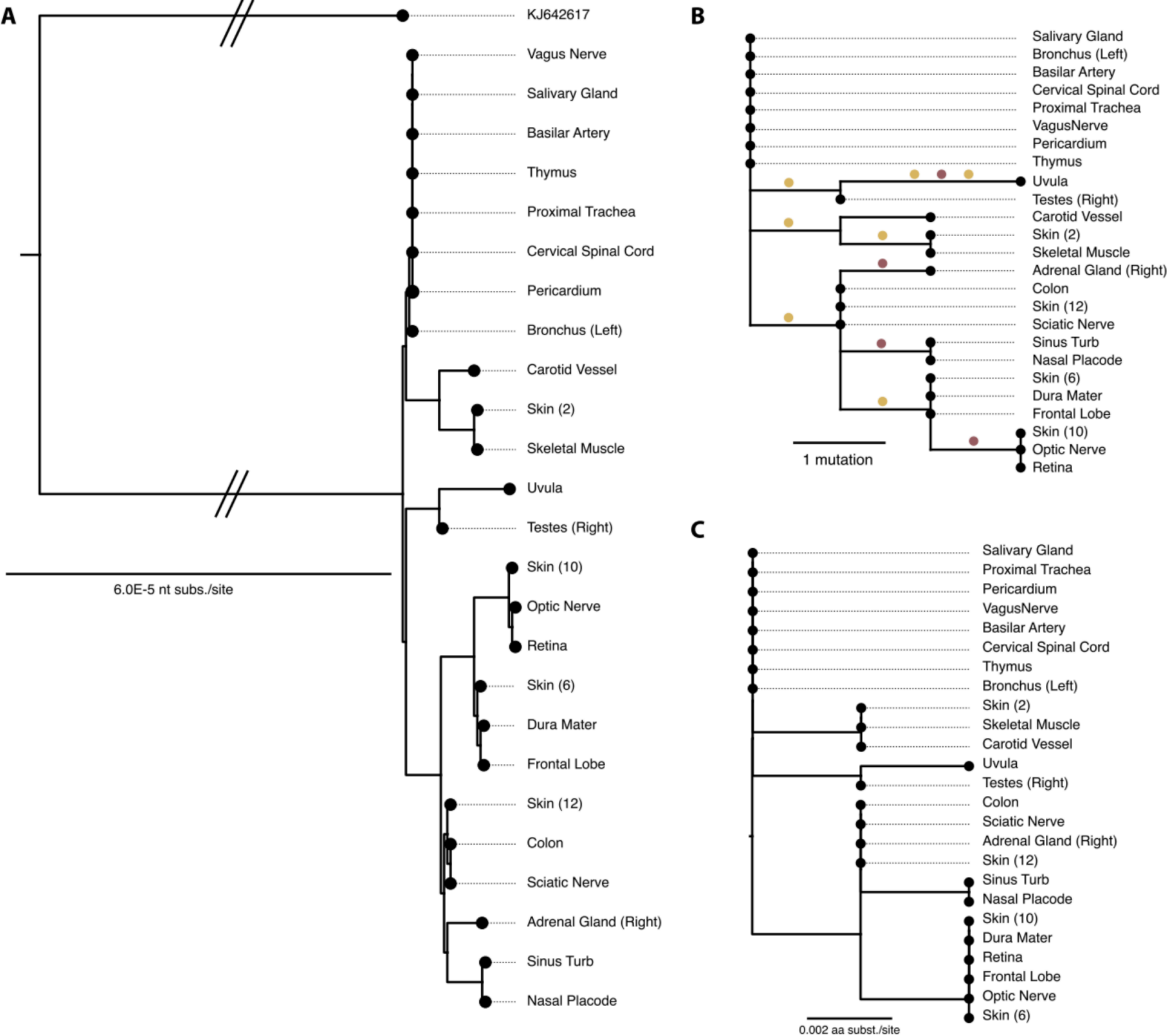
Within-host MPXV sequence phylogenetic analyses. **A.** Full genome maximum likelihood phylogenetic tree rooted using MPXV Clade II sequence KJ642617 (a canonical Clade IIb reference). Scale bar indicates nucleotide substitutions per site. **B.** Ancestral reconstruction performed for each internal node on the phylogeny of all sequences using IQ-TREE 2, enabling mapping of single nucleotide polymorphisms (SNPs) along branches. SNPs are colored by whether they are consistent with APOBEC3 deamination (red) or not (orange), scale bar indicates number of mutations per site. **C.** VP37 amino-acid maximum-likelihood phylogenetic tree, scale bar indicates amino acid substitutions per site. sub/site: substitutions per site. Skin (10), desquamated lesion; Skin (6), crusted lesion; Skin (2), hypopigmented scar; Skin (12), normal appearing/intact skin.

25 within-host full length consensus MPXV genomes were generated and assigned to clade IIb lineage B.1.2 by Nexclade (https://clades.nextstrain.org/). Maximum-likelihood phylogenetic analysis of the full genomes, rooted on KJ642617 (a canonical Clade IIb reference), produced a well-resolved tree with short branch lengths, indicating limited within-host divergence (Fig. 2A). No distinct tissue-specific clustering was observed consistent with widespread dissemination of a single systemic lineage. The APOBEC3-based tree showed a limited number of mutations, with samples from the same body system interleaved rather than forming monophyletic clades (Fig. 2B), a pattern indicating that APOBEC3-mediated editing acts broadly across tissues rather than in a compartment-specific manner.

The VP37 amino acid sequence revealed that tecovirimat resistance–associated mutations (A288P, A290V, I372N) were detected in multiple tissues but not universally across all samples (Table 1, Fig. 2C). Some sequences harbored single substitutions, while others carried two resistance-linked changes.

## Discussion

Here we report autopsy findings from a prolonged case of MPXV clade IIb infection in a person with advanced HIV and limited immune reconstitution, despite HIV viral load suppression, on ART. Over the course of more than seven months, the patient experienced progressive and destructive mucocutaneous disease, contributing to clinical deterioration and death. We detected the highest burden of MPXV DNA and replication-competent virus in the skin, oral, and nasopharyngeal tissues, consistent with known modes of MPXV transmission through direct contact with infected skin or mucosal lesions. We detected moderate amounts of MPXV DNA in visceral organs, with only sporadic replication-competent virus detected in liver and skeletal muscle. Despite detecting relatively lower amounts of MPXV DNA in CNS tissue, we confirmed replication-competent virus in olfactory nerve, frontal lobe, medulla, and midbrain, perhaps due to CNS being an immune privileged site, and validated MPXV infection of neurons and glial cells by ISH.

Neurological findings in patients with mpox are well described with headache and fatigue reported in approximately 30% of cases.^12^ More severe neurological findings in immune competent and immunocompromised patients, including encephalitis and encephalomyelitis, occur less commonly but are well documented with supportive CNS imaging with or without MPXV DNA detected in CSF.^13^ MPXV protein has been previously detected in microglia, implicating direct CNS infection,^14^ although no prior studies have confirmed replication-competent virus in CNS or neuronal infection.

Our patient did not exhibit overt signs or symptoms of encephalitis and histological evaluation of brain tissue did not reveal pathological changes or leukocytic infiltrate consistent with encephalitis. These observations indicate that MPXV can infect, replicate, and persist in the CNS of patients with advanced HIV. Since viremia occurs early following MPXV exposure and mild to severe neurological manifestations have been described in immunocompetent individuals with mpox, seeding of CNS may also occur in some immunocompetent patients. Mpox case-fatality ratio (CFR) correlates with CD4 count with CFR approximating 30% in patients with CD4< 100 cells/mm^3^.^2^ Consequently, the primary therapeutic intervention, beyond supportive care, in patients living with advanced HIV and mpox, is ART. However, immune reconstitution inflammatory syndrome (IRIS) has been described in up to 25% of people with advanced HIV and mpox who initiated or re-initiated ART, with 57% mortality observed in this cohort. Consistent with this, inflammatory necrotic lesions in visceral organs have been observed in such patients at autopsy.^3^ By contrast, we did not observe visceral necrotic lesions in our patient, perhaps because he achieved only limited immune reconstitution.

Randomized clinical trials indicate that mpox treatment with tecovirimat is not effi cacious.^15, 16^ Lack of effi cacy might be attributable to delayed administration or development of intra-host tecovirimat resistant variants.^7,8^ In our case we detected VP37 resistance–associated substitutions (A288P, A290V, I372N) in multiple tissues indicating that variants can emerge during or after tecovirimat exposure and persist. The heterogeneous distribution of these mutations suggests the presence of mosaic viral subpopulations within the host, supporting the notion that antiviral selection pressures, rather than tissue-specific adaptation, dominate intra-host evolution.

Since the end of the smallpox vaccination and eradication campaign in 1980,^17^ which provided cross-protection from mpox, the mpox-susceptible population has grown, underlying recent global spread. In addition to more than 100,000 global cases of MPXV clade IIb since 2022, primarily occurring among men who have sex with men, outbreaks of clade Ia and Ib currently burden central and west Africa.^18^ Many countries in these regions are experiencing sustained human-to-human transmission with patterns of spread expanded to heterosexual sex and close contact in households and healthcare settings.^17^ Meanwhile, a growing list of countries in Europe, the Americas, Asia, the Middle East, and Oceania have reported travel-associated clade Ib cases,^1^ and local community transmission of clade Ib was reported in California in August of 2025.^18^ These trends demonstrate increasing global mpox circulation with rising morbidity and mortality. Our case demonstrates potential for MPXV CNS infection, replication, drug resistance, and long-term persistence. Enhanced detection and control efforts and effective therapeutic development are urgently needed to limit the growing public health and economic burden of this disease.

## Supplementary Material

Supplementary Files

This is a list of supplementary files associated with this preprint. Click to download.

- Mpoxsupplement032026.docx

## Figures and Tables

**Table 1:** Tecovirimat resistance-associated mutations in the *F13L* gene (VP37 protein) homolog (OPG057).

	Nonresistance-associated positions		Resistance-associated positions		
Tissue	243 (allele frequency*)	259 (allele frequency)	288 (allele frequency)	290 (allele frequency)	372 (allele frequency)
**Skin (10)**	P	N	A	V (99)	N (99)
**Skin (6)**	P	N	A	V (88)	N (99)
Uvula	P	D (52)	A	V (53)	I
Skin (2)	P	N	P (85)	A	I
Salivary Gland	P	N	A	A	I
**Optic Nerve**	P	N	A	V (73)	N (77)
Colon	P	N	A	A	N (78)
Bronchus (Left)	P	N	A	A	I
Skin (12)	P	N	A	A	N (52)
Carotid Vessel	P	N	P (66)	A	I
Sciatic Nerve	P	N	A	A	N (54)
**Dura Mater**	P	N	A	V (50)	N (62)
Sinus Turb	S (76)	N	A	A	N (90)
Basilar Artery	P	N	A	A	I
Cervical Spinal Cord	P	N	A	A	I
Proximal Trachea	P	N	A	A	I
**Retina**	P	N	A	V (81)	N (84)
Nasal Placode	S (88)	N	A	A	N (95)
Vagus Nerve	P	N	A	A	I
Skeletal Muscle	P	N	P (57)	A	I
Pericardium	P	N	A	A	I
Thymus	P	N	A	A	I
**Frontal Lobe**	P	N	A	V (52)	N (52)
Adrenal Gland (Right)	P	N	A	A	N (70)
Testes (Right)	P	N	A	V (51)	I

Tecovirimat resistance-associated mutations are indicated in red. Sequences with more than 2 Tecovirimat resistance-associated mutations are bold and sequences without Tecovirimat resistance-associated mutations are greyed.

* Alelle frequency = proportion of mapped reads containing the corresponding mutation at each site. Skin (10), desquamated lesion; Skin (6), crusted lesion; Skin (2), hypopigmented scar; Skin (12), normal appearing/intact skin.
